# Family history of cancer, Ashkenazi Jewish ancestry, and pancreatic cancer risk

**DOI:** 10.1038/s41416-019-0426-5

**Published:** 2019-03-14

**Authors:** Tsuyoshi Hamada, Chen Yuan, Matthew B. Yurgelun, Kimberly Perez, Natalia Khalaf, Vicente Morales-Oyarvide, Ana Babic, Jonathan A. Nowak, Douglas A. Rubinson, Marios Giannakis, Kimmie Ng, Peter Kraft, Meir J. Stampfer, Edward L. Giovannucci, Charles S. Fuchs, Shuji Ogino, Brian M. Wolpin

**Affiliations:** 1 Department of Oncologic Pathology, Dana-Farber Cancer Institute and Harvard Medical School, 450 Brookline Avenue, Boston, MA 02215 USA; 2 Department of Medical Oncology, Dana-Farber Cancer Institute and Harvard Medical School, 450 Brookline Avenue, Boston, MA 02215 USA; 3 Department of Epidemiology, Harvard T.H. Chan School of Public Health, 677 Huntington Avenue, Boston, MA 02115 USA; 4 Division of Gastroenterology, Hepatology, and Endoscopy, Brigham and Women’s Hospital and Harvard Medical School, 75 Francis Street, Boston, MA 02115 USA; 5 Program in MPE Molecular Pathological Epidemiology, Department of Pathology, Brigham and Women’s Hospital and Harvard Medical School, 75 Francis Street, Boston, MA 02115 USA; 6 Broad Institute of Massachusetts Institute of Technology and Harvard, 415 Main Street, Cambridge, MA 02142 USA; 7 Department of Medicine, Brigham and Women’s Hospital and Harvard Medical School, 75 Francis Street, Boston, MA 02115 USA; 8 Department of Biostatistics, Harvard T.H. Chan School of Public Health, 677 Huntington Avenue, Boston, MA 02115 USA; 9 Channing Division of Network Medicine, Department of Medicine, Brigham and Women’s Hospital and Harvard Medical School, 181 Longwood Avenue, Boston, MA 02115 USA; 10 Department of Nutrition, Harvard T.H. Chan School of Public Health, 677 Huntington Avenue, Boston, MA 02115 USA; 11 Yale Cancer Center, 333 Cedar Street, New Haven, CT 06510 USA; 12 Department of Medicine, Yale School of Medicine, 333 Cedar Street, New Haven, CT 06510 USA; 13 Smilow Cancer Hospital, 20 York Street, New Haven, CT 06519 USA

**Keywords:** Risk factors, Cancer epidemiology, Pancreatic cancer

## Abstract

**Background:**

Individuals with a family history of cancer may be at increased risk of pancreatic cancer. Ashkenazi Jewish (AJ) individuals carry increased risk for pancreatic cancer and other cancer types.

**Methods:**

We examined the association between family history of cancer, AJ heritage, and incident pancreatic cancer in 49 410 male participants of the prospective Health Professionals Follow-up Study. Hazard ratios (HRs) were estimated using multivariable-adjusted Cox proportional hazards models.

**Results:**

During 1.1 million person-years (1986–2016), 452 participants developed pancreatic cancer. Increased risk of pancreatic cancer was observed in individuals with a family history of pancreatic (HR, 2.79; 95% confidence interval [CI], 1.28–6.07) or breast cancer (HR, 1.40; 95% CI, 1.01–1.94). There was a trend towards higher risk of pancreatic cancer in relation to a family history of colorectal cancer (HR, 1.21; 95% CI, 0.95–1.55) or AJ heritage (HR, 1.29; 95% CI, 0.94–1.77). The risk was highly elevated among AJ men with a family history of breast or colorectal cancer (HR, 2.61 [95% CI, 1.41–4.82] and 1.92 [95% CI, 1.05–3.49], respectively).

**Conclusion:**

Family history of pancreatic cancer was associated with increased risk of this malignancy. Family history of breast or colorectal cancer was associated with the increased risk among AJ men.

## Background

Pancreatic cancer is the third leading cause of cancer-related mortality in the United States (U.S.) with an overall 5-year survival rate of <10%.^1^ This high mortality rate is largely related to the diagnosis of patients at advanced stages when the disease is unresectable and thus incurable.^2^ Therefore, there is a great need for identification of risk factors for pancreatic cancer and establishment of screening strategies to reduce mortality associated with this malignancy.

Individuals with a family history of pancreatic cancer carry increased risks of pancreatic cancer^3–8^ and other cancer types.^9–11^ In addition to environmental and lifestyle factors shared by family members, studies have identified genes that contribute to familial clustering of pancreatic cancer, including *ATM*, *BRCA1*, *BRCA2*, *CDKN2A*, and *PALB2*, as well as *PRSS1* and *SPINK1* for hereditary pancreatitis.^12–18^ Evidence also links family history of other cancer types to increased risk of pancreatic cancer.^5–8,18,19^ *BRCA1* and *BRCA2* for hereditary breast and ovarian cancer, DNA mismatch repair genes responsible for Lynch syndrome, and *CDKN2A* for familial atypical multiple melanoma and mole syndrome may contain underlying germline mutations that predispose to pancreatic cancer and other cancer types.^16–23^ However, the association of family history of various cancer types with risk of pancreatic cancer has not been fully evaluated in prospective studies with long duration of follow-up. Furthermore, individuals of Ashkenazi Jewish (AJ) heritage represent a genetically distinct population,^24–27^ which is characterised by higher prevalence of germline mutations in *BRCA1*, *BRCA2*, *MSH2*, and *MSH6*,^28–30^ and higher incidence of various malignancies including pancreatic cancer.^31–35^ The National Comprehensive Cancer Network (NCCN) guidelines recommend genetic counselling for AJ individuals diagnosed with pancreatic cancer.^36^ Yet, the association of family history with risk of pancreatic cancer is not well defined in the context of religious heritage.

To test the hypothesis that family history of other cancer types is associated with increased risk of pancreatic cancer, we conducted a prospective study based on a large U.S. cohort. We further examined pancreatic cancer risk associated with cancer family history by AJ ancestry.

## Materials and methods

### Study population

We collected data from an ongoing prospective cohort study in the U.S., the Health Professionals Follow-up Study (HPFS).^37^ In 1986, the HPFS cohort was established when 51,529 male health professionals aged 40–75 years (dentists, optometrists, osteopaths, pharmacists, podiatrists, and veterinarians) returned a baseline questionnaire. Participants have been sent questionnaires to report demographics, medical and social history, and health outcomes such as cancer diagnoses every 2 years, and to report dietary patterns every 4 years. Participants were followed until diagnosis of pancreatic adenocarcinoma, death from any cause, or the end of follow-up (31 January 2016), whichever came first. The follow-up rate has been more than 90% for each follow-up questionnaire cycle. Informed consent was obtained from all participants at study enrolment, and the HPFS was approved by the institutional review board at Harvard T.H. Chan School of Public Health (Boston, MA, USA).

Patients with pancreatic cancer were identified by self-report, next-of-kin, or review of the National Death Index. Physicians blinded to exposure data confirmed the diagnosis of pancreatic adenocarcinoma by review of medical records, death certificates, or cancer registry data. Deaths were ascertained based on information from next of kin, U.S. postal service, and/or the National Death Index; this method has been shown to capture >98% of deaths.^38^ We excluded participants with a history of cancer at baseline (exception of non-melanoma skin cancer) and those without available data on family history of cancer.

### Assessment of family history of cancer

We utilised data on family history of cancer in first-degree relatives including parents, siblings, and offspring which have been prospectively collected from questionnaires. Family history was assessed in 1996 for breast cancer; in 1986, 1990, 1992, 1996, 2008, and 2012 for colorectal cancer; in 2008 for pancreatic cancer; in 1990, 1992, and 2008 for melanoma; in 1990, 1992, and 1996 for prostate cancer; and in 1992 for lung cancer. A positive family history for each cancer type was defined when the participant reported any first-degree relative diagnosed with the corresponding cancer. Participants were treated as those with a family history of a specific cancer type from the first report of a first-degree relative diagnosed with the cancer. Follow-up time for the analysis of each cancer type started at the first year when a family history of the cancer was assessed.

### Ascertainment of covariates

In the questionnaire from 1998, participants were asked their religious heritage (Ashkenazi Jewish, Sephardic Jewish, Catholic, Protestant, other Christian, Eastern, Muslim, or other). For analyses adjusted for or stratified by AJ ancestry, follow-up time started from 1998. Data on other covariates were collected at baseline in 1986 and in follow-up questionnaires, including age, race, smoking status, diabetes mellitus, body mass index (BMI), physical activity level, alcohol intake, and regular multivitamin use.

### Statistical analysis

Using the Cox proportional hazards regression model, we examined the association between family history of each cancer type and risk of pancreatic cancer, and estimated hazard ratios (HRs) of pancreatic cancer incidence and 95% confidence intervals (CIs). The multivariable Cox regression models were adjusted for the following potential confounders including known risk factors for pancreatic cancer:^2,39^ age (continuous), race (white, black, other, or unknown), calendar year of questionnaire cycle (continuous), smoking in pack-years (never, 0.1–4.9, 5–19.9, 20–39.9, or ≥40), history of diabetes (yes/no), body mass index (<25, 25–29.9, 30–34.9, or ≥35 kg/m^2^), physical activity (metabolic equivalent of task-hours/week in quintiles), alcohol intake (0, 0.1–4.9, 5–14.9, 15–29.9, or ≥30 g/day), and regular multivitamin use (yes/no). We further adjusted for religious heritage (AJ or others). These covariates were treated as time-dependent to account for changes over time, if applicable. To reduce intra-individual variation and to consider long-term influences, we used the cumulative average for relevant covariates, which was the mean of all available data prior to each questionnaire cycle. The assumption of proportional hazards for the exposure variables and covariates was verified using Schoenfeld residual plots, which supported the proportionality of hazards during the follow-up period (data not shown). We assessed statistical interaction by entering main effect terms and the cross-product of family history of cancer and a stratification variable into the model and evaluating the Wald test. In all analyses, 2-sided *P* values < 0.05 were considered statistically significant, and SAS statistical software (version 9.4, SAS Institute, Cary, NC, USA) was used.

## Results

During follow-up of 49,410 study participants (1,173,906 person-years), 452 men developed incident pancreatic adenocarcinoma. Table 1 summarises age-standardised baseline characteristics of participants, overall and by AJ ancestry. Supplementary Table S1 shows the distribution of family history of cancer by AJ ancestry. Compared with non-AJ men, AJ men were more likely to have family members with colorectal, breast, or pancreatic cancer, and were less likely to have those with prostate cancer. Compared to non-AJ individuals, AJ individuals appeared to be at a higher risk of pancreatic cancer, but the association did not reach statistical significance (multivariable-adjusted HR, 1.29; 95% CI, 0.94–1.77).

**Table 1:** Age-standardised characteristics of participants in the Health Professionals Follow-up Study, at baseline (1986) and by Ashkenazi Jewish ancestry (1998)

Characteristic^a^	Baseline	Ashkenazi Jewish^b^	
		No	Yes
No. of participants	49,410	29,985	5580
Age, years	54.5 (9.8)	65.0 (9.1)	67.0 (9.9)
Race			
White	90.7%	91.6%	90.8%
Black	1.0%	0.8%	0%
Other	3.3%	2.7%	3.8%
Unknown	5.0%	4.8%	5.4%
Body mass index, kg/m²	25.5 (3.4)	25.8 (3.3)	25.4 (3.0)
Smoking status			
Current smoker	10.0%	5.3%	2.5%
Pack-years in ever smokers	13.4 (19.0)	12.8 (18.8)	11.5 (17.0)
History of diabetes	3.2%	7.4%	7.7%
Physical activity, MET-hours/week	18.8 (26.3)	27.7 (21.7)	26.5 (21.3)
Alcohol intake, g/day	11.3 (15.4)	11.6 (13.6)	6.9 (8.6)
Regular multivitamin use	41.7%	59.1%	58.8%

*MET* Metabolic equivalent of task

^a^All variables other than age were standardised to age distribution of the study population. Mean (standard deviation) was presented for continuous variables

^b^Religious heritage was assessed in 1998 and was reported by 72.0% of participants from the baseline population in 1986

Family history of breast cancer was associated with higher risk of pancreatic cancer (HR, 1.40; 95% CI, 1.01–1.94; Table 2). There was a trend towards higher risk of pancreatic cancer in relation to family history of colorectal cancer (HR, 1.21; 95% CI, 0.95–1.55; Table 2). When further adjusted for AJ ancestry, these associations remained largely unchanged (HR, 1.50 [95% CI, 1.07–2.12] for breast cancer; and HR, 1.27 [95% CI, 0.93–1.73] for colorectal cancer; Table 3). As expected, family history of pancreatic cancer was associated with higher risk of the disease (HR, 2.79; 95% CI, 1.28–6.07; Table 2). We observed no statistically significant association of family history of prostate cancer, melanoma, or lung cancer with pancreatic cancer risk (*P* > 0.49, Table 2).

**Table 2:** Risk of incident pancreatic cancer by family history of cancer

Family history	No. of cases	Person-years	HR for pancreatic cancer (95% CI)	
			Age-adjusted	Multivariable-adjusted^a^
Breast cancer				
Absent	195	435,771	1 (referent)	1 (referent)
Present	46	76,038	1.37 (0.99–1.90)	1.40 (1.01–1.94)
Colorectal cancer				
Absent	372	1,021,094	1 (referent)	1 (referent)
Present	80	152,732	1.20 (0.94–1.54)	1.21 (0.95–1.55)
Pancreatic cancer				
Absent	55	145,931	1 (referent)	1 (referent)
Present	8	7529	2.49 (1.17–5.33)	2.79 (1.28–6.07)
Melanoma				
Absent	283	646,495	1 (referent)	1 (referent)
Present	12	30,435	0.91 (0.51–1.62)	0.90 (0.50–1.60)
Prostate cancer				
Absent	285	665,289	1 (referent)	1 (referent)
Present	55	114,591	1.10 (0.82–1.47)	1.10 (0.82–1.48)
Lung cancer				
Absent	197	457,620	1 (referent)	1 (referent)
Present	29	55,818	1.12 (0.75–1.68)	1.15 (0.77–1.72)

*CI* Confidence interval, *HR* hazard ratio, *MET* metabolic equivalent of task

^a^The Cox proportional hazards regression models were adjusted for age (continuous), race (white, black, other, or unknown), calendar year of questionnaire cycle (continuous), smoking in pack-years (never, 0.1–4.9, 5–19.9, 20–39.9, or ≥40), history of diabetes (yes/no), body mass index (<25, 25–29.9, 30–34.9, or ≥35 kg/m^2^), physical activity (MET-hours/week in quintiles), alcohol intake (0, 0.1–4.9, 5–14.9, 15–29.9, or ≥30 g/day), and regular multivitamin use (yes/no)

**Table 3:** Risk of incident pancreatic cancer by family history of cancer with adjustment for Ashkenazi Jewish ancestry

Family history	No. of cases	Person-years	HR for pancreatic cancer (95% CI)	
			Age-adjusted	Multivariable-adjusted^a^
Breast cancer				
Absent	167	331,775	1 (referent)	1 (referent)
Present	43	57,475	1.49 (1.06–2.10)	1.50 (1.07–2.12)
Colorectal cancer				
Absent	207	417,949	1 (referent)	1 (referent)
Present	52	72,484	1.28 (0.94–1.74)	1.27 (0.93–1.73)
Pancreatic cancer				
Absent	52	109,930	1 (referent)	1 (referent)
Present	8	5681	2.76 (1.29–5.93)	3.05 (1.39–6.69)
Melanoma				
Absent	189	354,438	1 (referent)	1 (referent)
Present	9	17,796	0.97 (0.49–1.90)	0.96 (0.49–1.90)
Prostate cancer				
Absent	201	381,247	1 (referent)	1 (referent)
Present	37	68,343	1.01 (0.71–1.44)	1.03 (0.72–1.47)
Lung cancer				
Absent	138	267,933	1 (referent)	1 (referent)
Present	20	32,726	1.11 (0.69–1.80)	1.14 (0.70–1.85)

*CI* Confidence interval, *HR* hazard ratio, *MET* metabolic equivalent of task

^a^The Cox proportional hazards regression models were adjusted for age (continuous), race (white, black, other, or unknown), calendar year of questionnaire cycle (continuous), smoking in pack-years (never, 0.1–4.9, 5–19.9, 20–39.9, or ≥40), history of diabetes (yes/no), body mass index (<25, 25–29.9, 30–34.9, or ≥35 kg/m^2^), physical activity (MET-hours/week in quintiles), alcohol intake (0, 0.1–4.9, 5–14.9, 15–29.9, or ≥30 g/day), regular multivitamin use (yes/no), and AJ ancestry (yes/no)

We examined the association of family history of breast or colorectal cancer with pancreatic cancer risk in strata of AJ ancestry (Table 4). Family history of breast cancer was more strongly associated with pancreatic cancer risk in AJ individuals than in non-AJ Jewish individuals (*P*_interaction_ = 0.04). A similar differential association was observed for colorectal cancer, but the test for interaction was not statistically significant (*P*_interaction_ = 0.13). Compared with AJ individuals with no family history, those with a family history of breast and colorectal cancer had HRs of 2.61 (95% CI, 1.41–4.82) and 1.92 (95% CI, 1.05–3.49), respectively. The association of family history of colorectal cancer with pancreatic cancer risk appeared to be stronger for never smokers than forever smokers. We observed no statistically significant effect modification for the association of family history of breast or colorectal cancer with pancreatic cancer risk by BMI or DM status (*P*_interaction_ > 0.60, Table 5). Nevertheless, those participants with a history of diabetes mellitus and a family history of breast or colorectal cancer had notably higher risks of pancreatic cancer compared to participants with no diabetes and family history (HR, 3.09 [95% CI, 1.60–5.98] for breast cancer; and HR, 2.44 [95% CI, 1.45–4.09] for colorectal cancer).

**Table 4:** Risk of incident pancreatic cancer by family history of breast or colorectal cancer, stratified by Ashkenazi Jewish ancestry

	Family history of breast cancer		*P*_interaction_^b^	Family history of colorectal cancer		*P*_interaction_^b^
	Absent	Present		Absent	Present	
Ashkenazi Jewish						
No. of cases	30	15		36	17	
Person-years	50,651	10,172		62,891	13,042	
Age-adjusted HR (95% CI)	1 (referent)	2.57 (1.40–4.73)	0.04	1 (referent)	1.92 (1.06–3.49)	0.12
Multivariable HR (95% CI)^a^	1 (referent)	2.61 (1.41–4.82)	0.04	1 (referent)	1.92 (1.05–3.49)	0.13
Non-Ashkenazi Jewish						
No. of cases	137	28		171	35	
Person-years	281,124	47,303		355,058	59,442	
Age-adjusted HR (95% CI)	1 (referent)	1.22 (0.81–1.82)		1 (referent)	1.10 (0.76–1.59)	
Multivariable HR (95% CI)^a^	1 (referent)	1.22 (0.82–1.83)		1 (referent)	1.11 (0.77–1.60)	

*CI* Confidence interval, *HR* hazard ratio, *MET* metabolic equivalent of task

^a^The Cox proportional hazards regression models were adjusted for age (continuous), race (white, black, other, or unknown), calendar year of questionnaire cycle (continuous), smoking in pack-years (never, 0.1–4.9, 5–19.9, 20–39.9, or ≥40), history of diabetes (yes/no), body mass index (<25, 25–29.9, 30–34.9, or ≥35 kg/m^2^), physical activity (MET-hours/week in quintiles), alcohol intake (0, 0.1–4.9, 5–14.9, 15–29.9, or ≥30 g/day), and regular multivitamin use (yes/no)

^b^*P*_interaction_ was calculated using the Wald test for the cross-product of cancer family history (yes/no) and Ashkenazi Jewish ancestry (yes/no)

**Table 5:** Risk of incident pancreatic cancer by family history of breast or colorectal cancer, stratified by covariates

	Family history of breast cancer		*P*_interaction_^b^	Family history of colorectal cancer		*P*_interaction_^b^
	Absent	Present		Absent	Present	
Never smoker			0.87			0.05
No. of cases	78	20		131	37	
Person-years	206,719	36,090		471,348	69,793	
Multivariable HR (95% CI)^a^	1 (referent)	1.39 (0.84–2.28)		1 (referent)	1.56 (1.07–2.26)	
Ever smoker						
No. of cases	115	24		234	39	
Person-years	213,983	37,221		513,042	77,773	
Multivariable HR (95% CI)^a^	1.10 (0.81–1.50)	1.45 (0.90–2.32)		1.35 (1.08–1.68)	1.27 (0.88–1.82)	
Body mass index < 25 kg/m^2^			0.77			0.87
No. of cases	85	20		159	35	
Person-years	192,917	34,310		450,009	66,556	
Multivariable HR (95% CI)^a^	1 (referent)	1.32 (0.81–2.17)		1 (referent)	1.24 (0.86–1.80)	
Body mass index ≥ 25 kg/m^2^						
No. of cases	110	26		213	45	
Person-years	242,735	41,707		567,445	85,899	
Multivariable HR (95% CI)^a^	1.00 (0.74–1.34)	1.46 (0.93–2.30)		0.98 (0.79–1.22)	1.17 (0.84–1.64)	
No diabetes			0.61			0.75
No. of cases	157	36		304	64	
Person-years	390,110	68,593		938,865	138,689	
Multivariable HR (95% CI)^a^	1 (referent)	1.34 (0.93–1.94)		1 (referent)	1.19 (0.90–1.56)	
Diabetes						
No. of cases	38	10		68	16	
Person-years	45,661	7445		82,229	14,043	
Multivariable HR (95% CI)^a^	1.87 (1.29–2.72)	3.09 (1.60–5.98)		1.86 (1.41–2.44)	2.44 (1.45–4.09)	

*CI* Confidence interval, *HR* hazard ratio, *MET* metabolic equivalent of task

^a^The Cox proportional hazards regression models were adjusted for the following covariates except for the stratification variable: age (continuous), race (white, black, other, or unknown), calendar year of questionnaire cycle (continuous), smoking in pack-years (never, 0.1–4.9, 5–19.9, 20–39.9, or ≥40), history of diabetes (yes/no), body mass index (<25, 25–29.9, 30–34.9, or ≥35 kg/m^2^), physical activity (MET-hours/week in quintiles), alcohol intake (0, 0.1–4.9, 5–14.9, 15–29.9, or ≥30 g/day), and regular multivitamin use (yes/no)

^b^*P*_interaction_ was calculated using the Wald test for the cross-product of cancer family history (yes/no) and a stratification variable

## Discussion

Within a large U.S. cohort with 30 years of follow-up, we prospectively examined the association of family history of cancer with risk of incident pancreatic cancer. In addition, we examined how the association differed according to religious heritage and major risk factors for pancreatic cancer. Specifically, we evaluated family history of melanoma and breast, colorectal, lung, pancreatic, or prostate cancer. Notably, family history of breast cancer was associated with approximately 40% higher risk of developing pancreatic cancer over the study follow-up period. Interestingly, the elevated risk with a family history of breast or colorectal cancer was largely confined to individuals of AJ descent, identifying a group of participants at particularly elevated risk of pancreatic cancer. Furthermore, in stratified analyses, participants with both diabetes mellitus and a family history of breast or colorectal cancer had a greater than 2-fold risk of developing pancreatic cancer. Similar to data from previous studies,^3–8^ we noted a 2.8-fold increase in risk for pancreatic cancer with a family history of this malignancy. These findings indicate the potential of family history to identify individuals at elevated risk for pancreatic cancer, particularly when combined with additional factors such as AJ ancestry and a history of diabetes mellitus.

In a meta-analysis of 8 case–control studies and 1 prospective cohort study involving 6568 pancreatic cancer cases, the pooled relative risk for pancreatic cancer comparing positive vs. negative family history of this malignancy was 1.80 (95% CI, 1.48–2.12).^40^ Although increased risk of pancreatic cancer has been also reported among individuals with a family history of other cancer types, data from prospective studies are limited with inconsistent conclusions. In a prospective study of a U.S.-based cohort, family history of breast and colorectal cancer was associated with modestly increased pancreatic cancer mortality with HRs of 1.06 (95% CI, 0.97–1.15) and 1.12 (95% CI, 1.01–1.23), respectively.^8^ Data from a large cancer registry in Sweden suggested no significant increase in pancreatic cancer incidence associated with positive family history of breast, colon, and rectal cancer with standardised incidence ratios (SIRs) of 0.99 (95% CI, 0.75–1.27), 0.91 (95% CI, 0.66–1.24), and 1.34 (95% CI, 0.94–1.87), respectively.^5^ In a nested case–control study pooling 12 prospective cohort studies and a case–control study, family history of breast and colorectal cancer was not statistically significantly associated with pancreatic cancer risk (odds ratios, 1.21 [95% CI, 0.97–1.51] and 1.17 [95% CI, 0.93–1.47], respectively).^6^

Shared genetic architecture may contribute to familial predisposition to multiple cancer types. For pancreatic cancer, these alterations may include germline high penetrance mutations in the DNA double-strand break repair genes responsible for inherited breast and ovarian cancer (e.g., *BRCA1, BRCA2, and PALB2)* and the DNA mismatch repair genes responsible for Lynch syndrome (e.g., *MLH1*, *MSH2*, *MSH6*, and *PMS2*).^18–20,41–43^ The Breast Cancer Linkage Consortium reported the relative risk for pancreatic cancer of 2.26 (95% CI, 1.26–4.06) for *BRCA1* mutation carriers and 3.51 (95% CI, 1.87–6.58) for *BRCA2* mutation carriers.^16,17^ A prospective study reported a SIR for pancreatic cancer of 10.68 (95% CI, 2.68–47.70) among 446 carriers of a mismatch repair gene mutation.^20^ The aggregate of multiple low penetrance single nucleotide variants may also contribute to shared risk of multiple malignancies.^44^

Few prospective studies have evaluated the association of family history with risk of pancreatic cancer after considering religious heritage. In the current study, family history of breast and colorectal cancer appeared to be more strongly associated with risk of pancreatic cancer in AJ men than in non-AJ men. AJ individuals are at higher risk of several cancer types including pancreatic cancer.^31–34^ Ashkenazi Jews are known to have founder mutations in *BRCA1* (185_186delAG and 5382insC reported in 0.4% and 0.1%, respectively, of Ashkenazi women)^28^ and *BRCA2* (6174delT reported in 0.6% Ashkenazi women)^28^ underlying hereditary breast cancer.^28,29^ They also have founder mutations in *MSH2* (1906G>C reported in 0.44% Ashkenazi Jews with colorectal cancer)^45^ and *MSH6* (3959_3962delCAAG and 3984_3987dupGTCA reported in 0.11% and 0.30%, respectively, of Ashkenazi Jews with colorectal cancer)^46^ underlying Lynch syndrome.^30^ Those founder mutations may contribute to predisposition to pancreatic cancer. Therefore, family history of breast cancer in AJ men may be a surrogate for founder mutations in *BRCA1* and *BRCA2*, whereas family history of colorectal cancer potentially serves as a surrogate for founder mutations in *MSH2* and *MSH6*. Taken together, family history of breast or colorectal cancer in Ashkenazi Jews may mark a population with high risk of pancreatic cancer mediated through genetic alterations in these genes or others. Considering the joint risk estimates associated with a family history of breast or colorectal cancer, AJ ancestry, and diabetes status may assist in defining high-risk populations for disease screening.

Our study has notable strengths, including a prospective study design, large sample size with over 400 pancreatic cancer cases, and availability of religious heritage data. The data on family history and covariates were collected prospectively and updated repeatedly, which allowed us to rigorously adjust for potential confounders, to evaluate effect modification by other risk factors, and more importantly, to assess cancer family history without substantial recall bias, which can be a major problem when analysing family history data after a cancer diagnosis.^47,48^

The current study also has limitations that should be considered, including the availability of family history data for a select set of malignancies and self-reported information on family history. Nonetheless, misclassifications of family history status would likely have biased our findings towards the null hypothesis, as all participants were asked their family history prior to a diagnosis of pancreatic cancer. Though we cannot rule out the possibility of unmeasured confounding, our multivariable models included known and potential confounders associated with pancreatic cancer risk,^2,39^ and our findings did not differ substantially between age-adjusted and multivariable-adjusted models. We investigated family history of several cancer types, such that false positive results from multiple hypothesis testing are a possibility. However, strong a priori hypotheses are present for family history of breast and colorectal cancer, given the known founder mutations related to these cancer types in individuals of AJ descent. Finally, all participants were males in the current study, and therefore, our findings should be validated in female populations.

In conclusion, family history of breast cancer in one or more first-degree relatives was associated with increased incidence of pancreatic cancer in a large prospective cohort study of U.S. men. Individuals with a family history of colorectal cancer appeared to carry a modestly increased risk of pancreatic cancer. These associations were more pronounced among individuals of AJ descent and with a history of diabetes mellitus. Therefore, family history of other malignancies may be relevant for determining pancreatic cancer risk, particularly when combined with known risk factors for this disease.

## Supplementary information

Supplementary Table S1

## Data Availability

The datasets used and/or analysed during the current study are available from the corresponding author on reasonable request.
