# Supplementary material for: Family history of cancer, Ashkenazi Jewish ancestry, and pancreatic cancer risk
Source: Br J Cancer. 2019 Mar 14;120(8):848–54. doi: 10.1038/s41416-019-0426-5 (PMC6474278; doi:10.1038/s41416-019-0426-5)
Supplement: Supplementary file 1 — Supplementary Table S1 [file 41416_2019_426_MOESM1_ESM.docx]

Supplementary Table S1. Family history of cancer by Ashkenazi Jewish ancestry

|  | **Ashkenazi Jewish** | |  |
| --- | --- | --- | --- |
| Family history | No | Yes | *P*^a^ |
|  |  |  |  |
| **Breast cancer** |  |  | < 0.001 |
| Absent | 20 249 (85.6%) | 3 689 (83.1%) |  |
| Present | 3 396 (14.4%) | 752 (16.9%) |  |
|  |  |  |  |
| **Colorectal cancer** |  |  | < 0.001 |
| Absent | 25 579 (85.9%) | 4 571 (82.6%) |  |
| Present | 4 190 (14.1%) | 965 (17.4%) |  |
|  |  |  |  |
| **Pancreatic cancer** |  |  | 0.003 |
| Absent | 16 087 (95.3%) | 2 904 (94.0%) |  |
| Present | 798 (4.7%) | 185 (6.0%) |  |
|  |  |  |  |
| **Melanoma** |  |  | 0.78 |
| Absent | 19 454 (95.8%) | 3 771 (95.7%) |  |
| Present | 846 (4.2%) | 168 (4.3%) |  |
|  |  |  |  |
| **Prostate cancer** |  |  | < 0.001 |
| Absent | 23 009 (84.4%) | 4 474 (87.0%) |  |
| Present | 4 266 (15.6%) | 671 (13.0%) |  |
|  |  |  |  |
| **Lung cancer** |  |  | 0.51 |
| Absent | 16 175 (89.0%) | 3 117 (89.3%) |  |
| Present | 2 007 (11.0%) | 372 (10.7%) |  |
|  |  |  |  |

^a^ *P* value was calculated using the chi-square test.
